# Geographical variation in the heterogeneity of mutualistic networks

**DOI:** 10.1098/rsos.150630

**Published:** 2016-06-08

**Authors:** Shoko Sakai, Soeren Metelmann, Yukihiko Toquenaga, Arndt Telschow

**Affiliations:** 1 Center for Ecological Research, Kyoto University, Otsu 520-2113, Japan; 2 Institut für Evolution und Biodiversität, Westfälische Wilhelms-Universität Münster, 48149 Münster, Germany; 3 Faculty of Life and Environmental Sciences, University of Tsukuba, Ibaraki 305-8572, Japan

**Keywords:** pollination, seed dispersal, ecological network, degree distribution, specialization, network heterogeneity

## Abstract

Plant–animal mutualistic networks are characterized by highly heterogeneous degree distributions. The majority of species interact with few partner species, while a small number are highly connected to form network hubs that are proposed to play an important role in community stability. It has not been investigated, however, if or how the degree distributions vary among types of mutualisms or communities, or between plants and animals in the same network. Here, we evaluate the degree distributions of pollination and seed-dispersal networks, which are two major types of mutualistic networks that have often been discussed in parallel, using an index based on Pielou's evenness. Among 56 pollination networks we found strong negative correlation of the heterogeneity between plants and animals, and geographical shifts of network hubs from plants in temperate regions to animals in the tropics. For 28 seed-dispersal networks, by contrast, the correlation was positive, and there is no comparable geographical pattern. These results may be explained by evolution towards specialization in the presence of context-dependent costs that occur if plants share the animal species as interaction partner. How the identity of network hubs affects the stability and resilience of the community is an important question for future studies.

## 1. Introduction

Mutually beneficial interactions among species are ubiquitous in nature. They can take many forms of service–resource interactions such as pollination and seed-dispersal mutualisms, and resource–resource interactions including plant–mycorrhiza interactions. They are geographically and evolutionarily omnipresent, with mutualist partners found in various organisms and in all ecosystems [1]. Traditionally, mutualisms have been viewed as tightly coevolved interactions between a pair of species. However, accumulated evidence now makes it clear that highly specific, one-to-one relationships are rarely observed [2]. Instead, often dozens or even hundreds of species with different levels of specialization to their partners form complex networks of interdependence. How the complexity evolved, is maintained, and affects mutualistic interactions are fundamental questions in ecology, but we did not have the methodology to deal with this complexity until recently.

**Figure 1.**
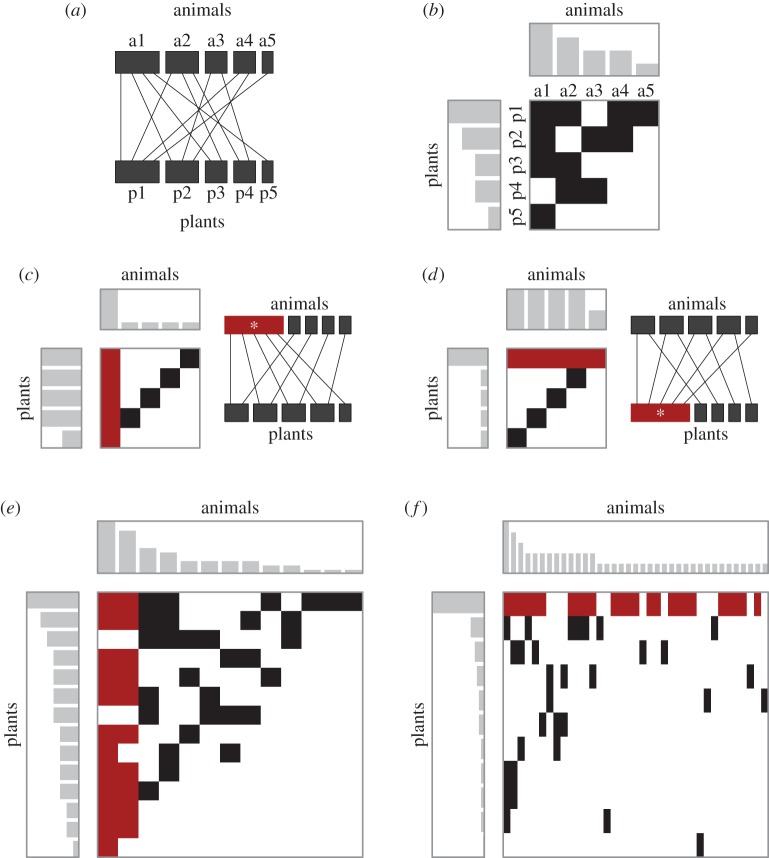
Plant–animal interaction networks. (*a*) An example of plant–animal interactions visualized as a bipartite graph. The networks formed by plant–animal mutualism are typically described as bipartite graphs, in which species of animals (a1–a5) interact with species of plants (p1–p5). (*b*) Interaction matrix of the network (*a*) with histograms showing degree distribution of plants (left) and animals (above). Black squares indicate interactions between plant and corresponding animal species. In the matrix and histograms, the plant and animal species are ranked in decreasing number of interactions per species (degree). In this network, degree distributions of plants and animals are identical (1 − *E*_P_ = 1 − *E*_A_ = 0.057). (*c*) An example of plant–animal interaction network with an animal hub. The hub species is indicated by a white asterisk. Degree distribution among animals is more heterogeneous (1 − *E*_A_ = 0.19) than that among plants (1 – *E*_P_ = 0.017). (*d*) A network with a plant hub. (*e*,*f*) Network representation of two actual pollination networks. (*e*) Flores, Azorean forest, Macronesia (P43 in electronic supplementary material, table S1) with an animal hub (1 − *E*_P_ = 0.039, 1 − *E*_A_ = 0.12), and (*f*) Llao Llao, Cerro López, Nahuel Huapi National Park in Rio Negro, Argentina (P37 in electronic supplementary material, table S1) with a plant hub (1 − *E*_P_ = 0.21, 1 − *E*_A_ = 0.038).

In the last two decades, network representation has emerged as an important tool for analysing complex ecological interactions, and several network characteristics are now well described [3–5]. The networks formed by plant–animal mutualisms, such as pollination and seed dispersal, are typically described as bipartite graphs, in which species in one taxonomic category, guild or trophic level, e.g. animals, interact with species in a second category, e.g. plants [3] (figure 1*a*). Analyses of empirical datasets have revealed several common characteristics of and variation among mutualistic networks such as nestedness, modularity, specialization and heterogeneity [3–6]. More recent studies have focused on spatial or temporal variations in these characteristics [7–10], mechanisms that are responsible for the patterns [11–14] and relationships among these characteristics [15].

In the network sciences, the degree distribution or the distribution of the number of links per species in the case of ecological networks, is a widely used measurement of the topology of complex networks. Degree distributions and their causes and consequences have been studied in both food webs and mutualistic networks [5,16–19]. Some mutualistic networks exhibit a power-law degree distribution (a decaying straight line in a log–log plot of cumulative number of species per degree category versus degree), while a majority exhibits a ‘truncated power-law’ (a straight line in a log–log plot with a sharp cut-off at high degree values [5]). In other words, the majority of species interact with few partner species, while a small number are highly connected [3,5]. These generalist species form network hubs and are proposed to play an important role in community stability [3] and to reduce the probability of secondary species extinction [20]. Comparatively less attention, however, has been paid to the questions whether the degree distribution differs between the two parties of a bipartite network, whether different types of mutualism differ with respect to their degree distribution, and whether there are differences between geographical regions.

In this study, we investigate the degree distribution of 56 pollination and 28 seed-dispersal networks. In bipartite networks of plants and animals, we do not have a reason to assume that the degree distributions of plants and animals change synchronously (figure 1). Therefore, we evaluated the degree distributions of the plant and animal sides separately, and tested for geographical variation. We then explored to what extent the observed patterns are explained by (i) variation in the degree of specialization and (ii) possible selective interactions between the species. For this analysis, we used the 28 pollination and 16 seed-dispersal datasets with quantitative measurements. To address (i), we examined changes in the degree distribution along with existing variation in the specialization among networks. To address (ii), we conducted a randomization analysis to investigate the extent to which the observed networks differed from those expected from random interactions of plants and animals.

## 2. Material and methods

### 2.1. Datasets

We compiled a total of 56 pollination and 28 seed-dispersal networks obtained from the literature and Web databases (electronic supplementary material, table S1). Datasets with fewer than eight animal or plant species were excluded from the analysis because they were likely to produce statistical artefacts. The networks were represented in binary matrix form, with the plant species in rows and the animal species in columns (figure 1*b*). The matrix elements were 1 when an interaction of a plant and an animal species was observed and 0 when interactions were absent. The datasets were categorized into the following five groups based on geographical region: (i) arctic and boreal (latitude > 55°); (ii) temperate (23° < latitude < 55°, altitude < 1600 m); (iii) tropics and subtropics (latitude < 23°, altitude < 1600 m); (iv) alpine (altitude > 1600) and (v) oceanic islands (New Zealand, Flores in Azores and Ile aux Aigrettes in Mauritius). For 28 pollination and 16 seed-dispersal datasets, quantitative measurements of interaction frequencies, such as number of animal visits per plant, were also available. They were used to evaluate network specialization and the selectivity of interactions (see below) and to examine robustness of the results (electronic supplementary material, figure S2).

### 2.2. Metrics used for degree distribution

We used an evenness index to evaluate the skewed degree distribution of links among plant and animal species (distribution of the number of interacting species among plants and animal species). An important requirement of the index for this study was robustness to variation in rare taxa, given the stochastic nature of the presence or absence of the least abundant species in the dataset. Some evenness indices change substantially when a single individual of a new taxon is added to a sample with a large number of individuals that are evenly distributed among species [21]. Beisel [21] found four evenness indices that satisfied the condition (electronic supplementary material, table S2).

The other critical index property is independence from sample sizes or species richness. This is because we used datasets that differed in sampling effort and species richness of the community, so the number of observed interactions varied by several orders of magnitude among datasets. In most cases, the evenness index is correlated with species richness, and the strength of the correlation depends on both the type of index and the relative species abundance (e.g. [22]). To assess the dependence of the four indices on species richness in measuring the link distribution, we plotted the indices against the number of species for plants and animals separately (electronic supplementary material, figure S1). Among the four, *E*_Pielou_ [23] and $E_{-\ln\;D}$ [24] showed no significant correlation with the number of species. Therefore, they satisfied the requirements of this study. We chose *E*_Pielou_ because of its popularity, although the two indices provided highly correlated values (for degree distribution among plant species, correlation coefficient = 0.99, *p* < 0.0001; for animals, correlation coefficient = 0.96, *p* < 0.0001).

Using the index *E*_Pielou_, the evenness of the degree distribution for plants (*E*_P_) and animals (*E*_A_) was calculated as follows:

$$E_{P}=\frac{-\sum_{i=1}^{N}x_{i}\cdot \ln(x_{i})}{\ln(N)}\;\mathrm{and}\;E_{A}=\frac{-\sum_{j=1}^{M}y_{j}\cdot \ln(y_{j})}{\ln(M)},$$

where *N* and *M* represent the numbers of plant or animal species, respectively, and *x_i_* and *y_j_* are the proportions of links that belong to the plant species *i* and animal species *j* relative to the total number of links in the network. The numerical evenness values are between 0 and 1, with 1 representing complete evenness. Because in this paper, we emphasize heterogeneity instead of evenness, we define the level of heterogeneity as 1−*E*_P_ and 1−*E*_A_, with values ranging from 0 to 1.

The metrics are robust against variation in sampling effort. We examined the possible dependence of 1−*E*_P_ and 1−*E*_A_ on sampling effort by randomization using the 44 networks with quantitative measurement (electronic supplementary material, table S1). We imitated a 50% reduction in sampling effort by randomly removing half of the visit records and calculating evenness. The procedures were repeated 1000 times for each network. The reduction of the samples caused a decrease in the number of plant and animal species in the networks by 9.7 ± 7.9% and 15.8 ± 11.2%, respectively, and a decrease of the links by 26.3 ± 9.6% (mean ± s.d. of 44 networks). However, the average evenness indices calculated for the reduced samples were highly correlated and did not show large deviations from the original values (electronic supplementary material, figure S2).

### 2.3. Metrics used for network specialization

Blüthgen *et al*. [25] introduced a quantitative index using interaction frequencies to describe the degree of specialization, based on information theory. This measure ($H_{2}^{\prime }$) is derived from Shannon entropy and characterizes the level of specialization of the entire network. The metric is standardized and ranges from 0 for the most generalized to 1 for the most specialized network. Because we aimed at comparing the most unbiased estimates of network specialization, we used a double standardized specialization index $\Delta H_{2}^{\prime }=H_{2}^{\prime }-H_{2\mathrm{ran}}$, where *H*_2ran_ represents the mean $H_{2}^{\prime }$ of 1000 randomized networks (see [7,8]). Randomizations were performed with the Patefield algorithm, which randomly redistributes interaction events among all cells of the network while constraining total interaction strength per species. Therefore, the model assumes that partners associate randomly in the absence of any specialization. Calculation of $H_{2}^{\prime }$ and the Patefield algorithm were implemented as the function ‘H2fun’ and ‘r2dtable’, respectively, in the package ‘bipartite’ for R statistical software [26].

We note that the definition of ‘specialization’ of species or communities differs among authors and studies. While $\Delta H_{2}^{\prime }$ evaluates the deviation of the focal network from random interactions, other indices are based on the average numbers of interacting partner species [27,28] and network modularity [9].

### 2.4. Adjustment of plant–animal ratio

The numbers of plant and animal species varied greatly between pollination and seed dispersal as well as among networks. To examine whether the large variation in the ratios of plant and animal species substantially affects the results, we reduced the variation by the following procedure and repeated the analysis with the altered networks.

The log-transformed ratio of animal species to plant species ranged from −0.94 to 2.14 for pollination and −1.53 to 0.98 for seed dispersal. If this ratio was lower than 0.0 or higher than 1.0 for a given network, we randomly removed surplus plant or animal species from the network, respectively. The removal of plant species often made some animal species unconnected or vice versa, and we also removed the unconnected species from the network. We repeated the procedure 1000 times for each network and recorded averages of 1 − *E*_P_, 1 − *E*_A_ and plant–animal ratio. The procedure significantly reduced the standard deviation of the ratio from 0.72 to 0.33 among pollination and from 0.62 to 0.20 among seed-dispersal networks.

### 2.5. Randomization

We conducted a randomization analysis to investigate the extent to which the observed networks differed from those expected from random interactions of plants and animals using the 28 pollination and 16 seed-dispersal datasets with quantitative measurements (electronic supplementary material, table S1). For each dataset, we generated 1000 random networks with the same distribution of observation frequencies among species, and 1 − *E*_P_ and 1 − *E*_A_ of observed networks were compared with averages of those networks created by randomization. Again, the Patefield algorithm was used to generate random matrices.

## 3. Results

### 3.1. Variation of 1 − *E*_P_ and 1 − *E*_A_

**Figure 2.**
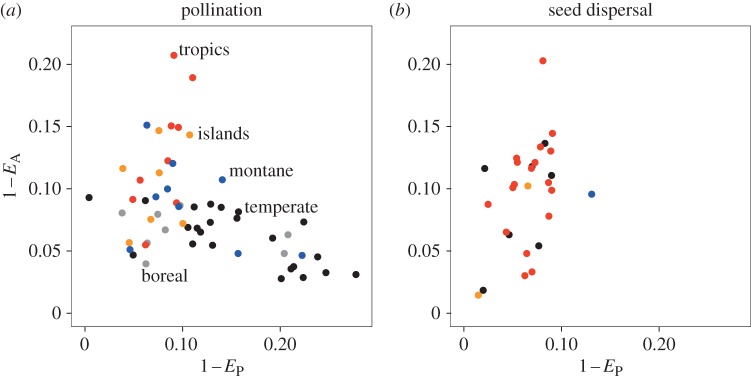
Relationships between 1 − *E*_P_ and 1 − *E*_A_ of pollination and seed dispersal. Geographical regions are distinguished by colour, as indicated in the figure.

1 − *E*_P_ and 1 − *E*_A_ of pollination networks are significantly different among geographical regions (results of Kruskal–Wallis rank sum test for 1 − *E*_P_, Kruskal–Wallis *χ*^2^ = 16.1, d.f. = 4, *p* = 0.0028; for 1 − *E*_A_, Kruskal–Wallis *χ*^2^ = 19.9, d.f. = 4, *p* = 0.0005), whereas we did not detect significant north–south differences. Temperate communities have higher 1 − *E*_P_ than 1 − *E*_A_, whereas the tropics and islands show the opposite pattern. As a result, the magnitude of the relationship between 1 − *E*_P_ and 1 − *E*_A_ changes among geographical regions (figure 2*a*). Significant geographical variation in 1 − *E*_P_ and 1 − *E*_A_ was not observed for seed dispersal (for 1 − *E*_P_, Kruskal–Wallis *χ*^2^ = 4.8, d.f. = 4, *p* = 0.19; 1 − *E*_A_, Kruskal–Wallis *χ*^2^ = 2.1, d.f. = 4, *p* = 0.55).

1 − *E*_P_ and 1 − *E*_A_ had strong negative correlation among pollination networks (figure 2*a*, *ρ* = −0.45, *p* = 0.0005, *n* = 56, Spearman's rank correlation test), and the relationship remained unchanged after adjustment of the ratio of animal to plant species (electronic supplementary material, figure S3*a, ρ* = −0.31, *p* = 0.018). On the other hand, the correlation between 1 − *E*_P_ and 1 − *E*_A_ was positive and significant in seed dispersal (figure 2*b, ρ* = 0.43, *p* = 0.018, *n* = 28), while the correlation was weaker and not statistically significant for adjusted networks (electronic supplementary material, figure S3*b*, *ρ* = 0.28, *p* = 0.149, for seed dispersal).

### 3.2. Change of *E*_P_ and *E*_A_ along specialization gradient

**Figure 3.**
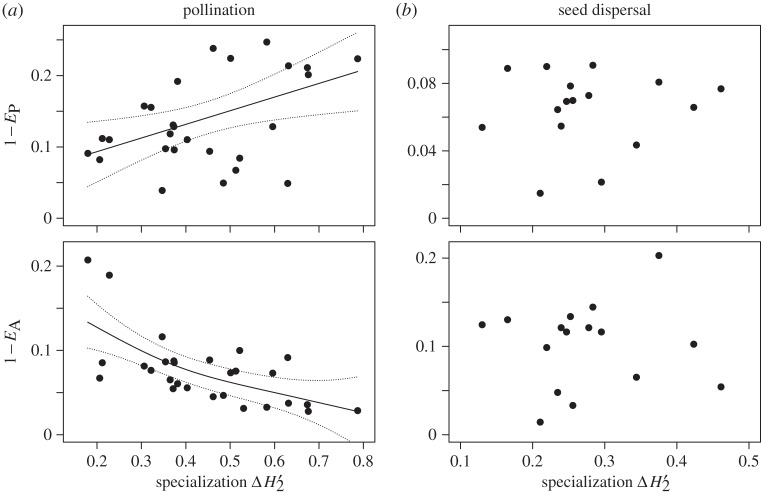
Changes of 1 − *E*_P_ and 1 − *E*_A_ along specialization gradient. (*a*) Pollination. The relationships were estimated by a generalized additive model, and the estimates and 95% confidential intervals are shown by solid and dotted lines, respectively. We used the mgcv package [29] implemented in R [26] for the additive model analysis. (*b*) Seed dispersal.

The specialization index $\Delta H_{2}^{\prime }$ of pollination matrices ranged from 0.18 to 0.79, while that of seed dispersal was much smaller and less variable ranging from 0.13 to 0.46. As for relationships between $\Delta H_{2}^{\prime }$ and the heterogeneity of degree distributions, we found that 1 − *E*_P_ significantly increased and 1 − *E*_A_ significantly decreased along the specialization gradient in pollination (figure 3*a*, for 1 − *E*_P_, *ρ* = 0.38, *p* = 0.047; for 1 – *E*_A_, *ρ* = −0.59, *p* = 0.0011; *n* = 28, Spearman's rank correlation test). On the other hand, no correlation was found for seed-dispersal networks (figure 3*b,* for 1 − *E*_P_, *ρ* = 0.10, *p* = 0.71; for 1 − *E*_A_, *ρ* = 0.01, *p* = 0.98, *n* = 16).

### 3.3. Comparison with random networks

**Figure 4.**
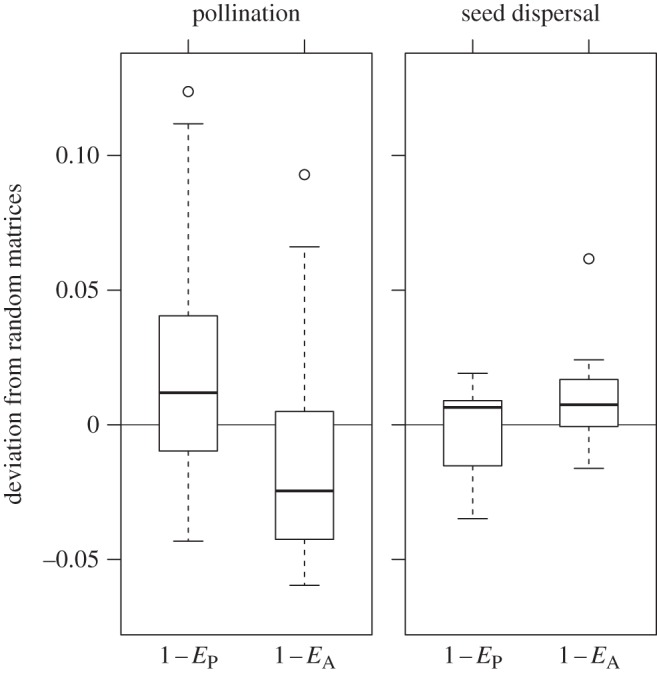
Deviation of 1 − *E*_P_ and 1 − *E*_A_ of original matrices from random ones. Thick horizontal lines are medians, bars indicate 25 and 95 percentiles, whiskers indicate the data range and the circles are outliers.

In pollination networks, differences of 1 − *E*_P_ and 1 − *E*_A_ of observed networks from those created by randomization ranged from −0.042 to 0.124 and from −0.060 to 0.093, respectively (figure 4). 1 − *E*_P_ was larger than that of random ones with marginal significance (Wilcoxon signed-rank test, *V* = 282 *p* = 0.074, *n* = 26) and 1 − *E*_A_ was significantly lower (Wilcoxon signed-rank test, *V* = 111, *p* = 0.036). In seed-dispersal networks, deviations were small compared with those of pollination networks (1 − *E*_P_, −0.035 to 0.019; 1 − *E*_A_, −0.016 to 0.061).

Significant negative correlation between 1 − *E*_P_ and 1 − *E*_A_ found in pollination networks (Spearman's rank correlation test, *ρ* = −0.68, *p* = 0.0001, electronic supplementary material, figure S4*a*) was not observed among randomly generated networks (*ρ* = 0.22, *p* = 0.27, electronic supplementary material, figure S4*c*). For seed dispersal, however, there is a positive correlation between 1 − *E*_P_ and 1 − *E*_A_ that is marginally significant for observed networks (*ρ* = 0.49 *p* = 0.054, *n* = 28, electronic supplementary material, figure S4*b*) and significant for random networks (*ρ* = 0.56, *p* = 0.026, electronic supplementary material, figure S4*d*).

## 4. Discussion

This study revealed remarkable differences between pollination and seed dispersal by investigating variation of the degree distributions of mutualistic networks. For pollination, we found a strong negative correlation between 1 − *E*_P_ and 1 − *E*_A_ (figure 2*a*). Temperate pollination communities are characterized by relatively even degree distribution in animals (low 1 − *E*_A_) and heterogeneous degree distribution in plants (high 1 − *E*_P_), whereas the tropics and islands show the opposite pattern (figure 2*a*). In other words, temperate pollination networks have ‘plant hubs’, whereas the tropics and islands have ‘animal hubs’. Absence of the negative correlations between 1 − *E*_P_ and 1 − *E*_A_ among random pollination networks (electronic supplementary material, figure S4) suggests that the negative correlation observed in networks is the result of selective processes. In contrast with pollination, we found no geographical differences among seed-dispersal networks, and the correlation between 1 − *E*_P_ and 1 − *E*_A_ was positive (figure 2*b*). The difference is remarkable, because these two major types of mutualistic networks are often discussed in parallel and their qualitative differences have rarely been reported. Our supplementary analysis indicates that the positive correlation in seed dispersal is due to some structural constraints, but the negative correlation in pollination is not (electronic supplementary material, note S1).

The results suggest that the structures of pollination and seed-dispersal networks are built by different ecological mechanisms. An important aspect of plant–pollinator interactions distinct from seed dispersal is that network structure strongly affects the fitness of individual plants. Pollinators that are specialized to a certain plant species have a high rate of conspecific pollen transport, whereas the efficiency of pollination is low for generalist pollinators that are shared by different plant species [30,31]. It may be advantageous for plants to exclude generalist pollinators, which have a low rate of conspecific pollen transfer. On the other hand, efficiency of seed delivery to suitable germination sites is not affected by the level of specialization of the dispersal agent [32]. This difference was previously suggested to be a reason of greater degrees of specialization in pollination networks than that of seed dispersal [6,32]. However, how specialization modifies degree distributions of mutualistic networks and if it differs between plants and animals, or between pollination and seed dispersal has not been studied yet.

To examine how specialization associates with the degree distributions, we examined changes in the degree distribution along the gradient of the network-level specialization. For pollination, we found that $\Delta H_{2}^{\prime }$ is negatively correlated to animal heterogeneity, 1 − *E*_A_, and positively correlated to plant heterogeneity, 1 − *E*_P_ (figure 3*a*). These results are difficult to explain, in particular, due to the limited knowledge of the processes that cause variation of specialization among communities. Here, we cautiously interpret the results from an evolutionary perspective. Let us consider a baseline community network with low levels of specialization, and let us assume that selection in this community favours plant species that interact with efficient pollinators, i.e. pollinators that are shared with few or no other plant species. In such a situation, we expect that evolution drives the network to higher levels of specialization, and that this increase is accompanied by decreasing 1 − *E*_A_. Such an evolutionary scenario may explain the negative correlation between $\Delta H_{2}^{\prime }$ and 1 − *E*_A_ if there is variation among communities in the strength of selection. On the contrary, the positive correlation between $\Delta H_{2}^{\prime }$ and 1 − *E*_P_ may be due to the difference in the advantage generalist and specialist plant species get from specialization (or exclusion of inefficient pollinators). For a generalist plant species pollinated by many animal species, the advantage from excluding an inefficient pollinator is low because the proportion of the pollen delivered by the pollinator species is small. On the other hand, for a specialist plant species, which only has a few pollinator species, the advantage of excluding an inefficient pollinator is large compared with a generalist species, as long as the plant receives a sufficient amount of pollinator visits. Obviously, such processes would promote the differentiation of generalist and specialist plant species, and, further, may explain why 1 − *E*_P_ increases with $\Delta H_{2}^{\prime }$. It is an open question whether such an evolutionary framework is responsible for the correlation pattern of $\Delta H_{2}^{\prime }$ with 1 − *E*_A_ and 1 − *E*_P_. Whatever the real cause, however, it suggests that network specialization is key to understand the negative correlation between 1 − *E*_A_ and 1 − *E*_P_. We remark that, unlike pollination, we did not find clear association of 1 − *E*_A_ and 1 − *E*_P_ with $\Delta H_{2}^{\prime }$ in the seed-dispersal networks (figure 3*b*). It is consistent with the supposition that plants do not have a preference for specialists or generalist seed dispersers because seed-dispersal efficiency is not directly related with the width of the food plants of the dispersal agents.

For pollination networks, we also explored how selective interactions relate to network heterogeneity using a randomization analysis. The null hypothesis in the analysis assumes that plants and animals randomly interact with each other without discriminating partners. The analysis allows to directly test how selective interactions modify the degree distribution for a given distribution of interactions among species. We found that the directions of the deviations are consistent with the direction of the changes in the former analysis. 1 − *E*_P_ of the original matrices was higher than that of random ones and 1 − *E*_A_ was lower (figure 4). This suggests that the correlations of 1 − *E*_P_ and 1 − *E*_A_ with $\Delta H_{2}^{\prime }$ are at least in part due to selective processes.

Given close relationships between the specialization and degree distributions, the geographical variation of 1 − *E*_P_ and 1 − *E*_A_ we found may arise from different network-level specialization among regions. Geographical variation in community-level specialization of plant–pollinator interactions and its ecological correlates are topics of long-standing interest [33,34], while attempts to quantify the variation have only recently begun [6,8,25,27,28,35]. On oceanic islands, biologists repeatedly found ‘super-generalist’ pollinators and much generalized interactions [36–38]. More recently, Schleuning *et al*. [8] have reported an increase in the specialization with the latitude both in pollination and seed dispersal, contrary to the notion that biological interactions are more specialized in species rich tropical community.

Variation in specialization in plant–pollinator networks has frequently been explained in connection with relative species richness or relative abundance of plants and pollinators. Generalized interactions on oceanic islands are often attributed to paucity of pollinator fauna [36]. Similarly, Schleuning *et al*. [8] suggest higher plant diversity in the lower latitudes as a cause of the generalized relationships; reduced densities of resource plants associated with species diversity may lead to longer search times during foraging and constrain the specialization of animals as predicted from optimal foraging theory [39]. There are also theoretical studies suggesting that insufficient pollinators or pollination prevent specialization to reduce the cost of pollinator sharing [40,41]. It is because specialization, i.e. exclusion of a part of the flower visitors, decreases the plant reproductive success if a part of the pollen remains undispersed at the end of flowering, even if the pollination efficiency of the excluded visitors is low.

Although geographical variation in pollinator abundance or pollination service availability has not been tested directly, there is circumstantial evidence that may indicate some differences between tropics and temperate, and between islands and continental regions. First, fruit set of tropical plants tend to be low compared with temperate plants [42], and reproduction is more pollination limited in the tropics and on islands compared with other regions [27,28]. In addition, frequency of vertebrate pollination is higher in the tropics and on islands [36,43,44]. It could be interpreted as adaptation by plants to pollinator shortages. Pollinator paucity might, therefore, be a common feature of the tropics and islands.

There are other environmental factors that could independently affect specialization and degree distributions. Phenology of plants and pollinators is suggested to be an important factor to determine the specialization level of pollination networks [45]. In temperate latitudes, active periods of the pollinators and flowering periods of the plants are often limited to a particular season. It may cause higher specialization at the network level in the higher latitudes [46]. In a tropical climate, highly social pollinators such as honeybees and stingless bees are abundant throughout the year and are the most important pollinators in the community [8,47], while only few plants flower continuously [48,49]. The contrast may explain why tropical networks have animal hubs. On the other hand, species richness of insect pollinators shows great seasonal fluctuation in seasonal climate (e.g. [50]). Plants that flower at the peak of pollinator activities may attract more diverse visitors than others and appear as network hubs in temperate communities.

## 5. Conclusion

Potential asymmetries between plants and animals have largely been overlooked in the studies of mutualistic networks, though effects of the network structure on the fitness of the two parties are often very different. In pollination, we found geographical shifts of network hubs between plants and animals, which are substantially different in the flexibility of partner choice and other ecological characteristics. Pollinator animals could actively choose and change plants to visit depending on environmental factors such as resource availability and competition with other species [51], while changes of pollinator fauna from plant side is possible only through evolutional changes in the floral characteristics [2]. How the identity of network hubs affects the stability and resilience of the community is an important question for our understanding of evolution and maintenance of inter-specific mutualisms as well as for the management of an essential ecosystem service of pollination.

## Supplementary Material

Figure S1. Correlations between the number of species and the four evenness indices.

## Supplementary Material

Figure S2. (a) 1–EP and (b) 1–EA of the networks with reduced samples plotted against the corresponding values of the original networks

## Supplementary Material

Figure S3. Relationships between 1–EP and 1–EA for the networks with adjusted plant-animal ratio of (a) pollination and (b) seed dispersal.

## Supplementary Material

Figure S4. Plots of 1–EP and 1–EA of original networks for (a) pollination and (b) seed dispersal and random networks for pollination (c) and (d) seed dispersal.

## Supplementary Material

Note S1. Comparison of observed and potential maximum values of 1–E.

## Supplementary Material

Table S1. List of the 56 plant-pollinator networks and 22 plant-seed disperser network analyzed by this study.

## Supplementary Material

Table S2. Four evenness indices that are robust to changes in rare species and that respond moderately to changes in abundance of median and dominant species identified by Beisel [21].

## Data accessibility

The datasets and R scripts supporting this article are available from Dryad Digital Repository (http://dx.doi.org/10.5061/dryad.kv09p).
