# Supplementary figures and images for: Geographical variation in the heterogeneity of mutualistic networks
Source: R Soc Open Sci. 2016 Jun 8;3(6):150630. doi: 10.1098/rsos.150630 (PMC4929896; doi:10.1098/rsos.150630)

Figure S1

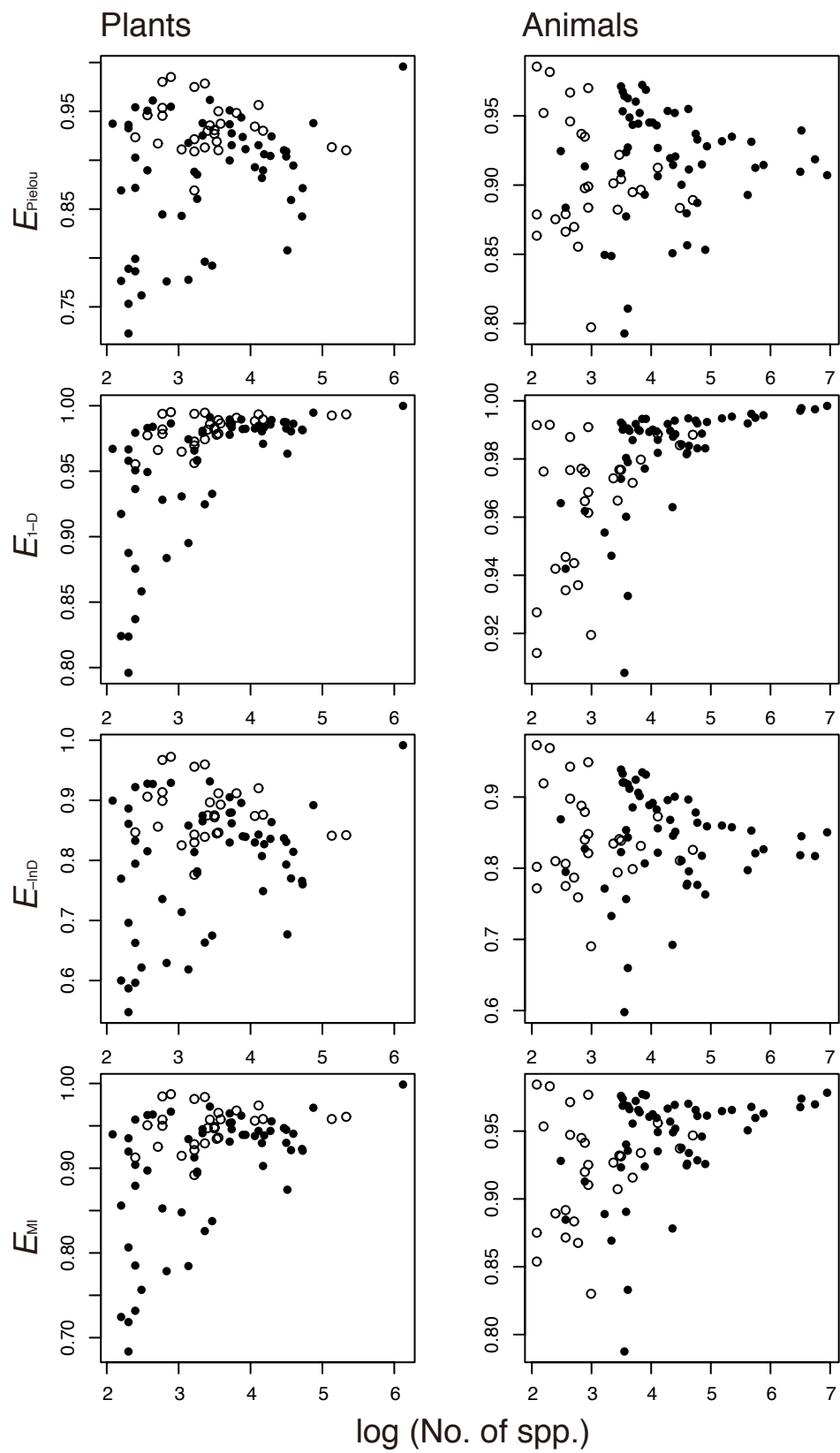

Supplement: Figure S1. Correlations between the number of species and the four evenness indices. [file rsos150630supp1.pdf]

Figure S2

(a)

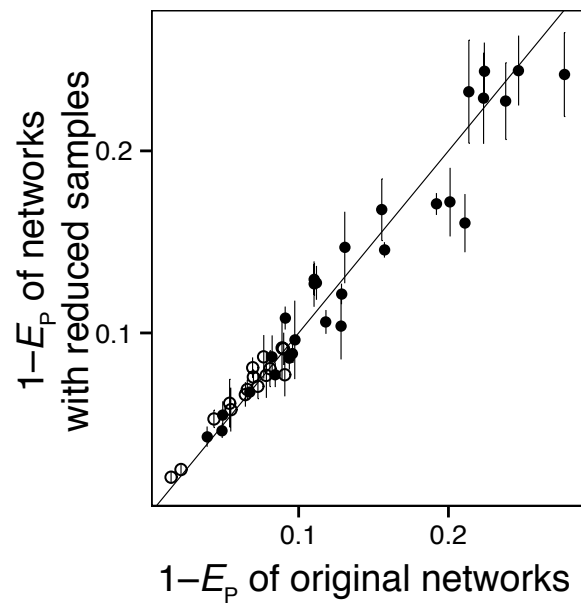

(b)

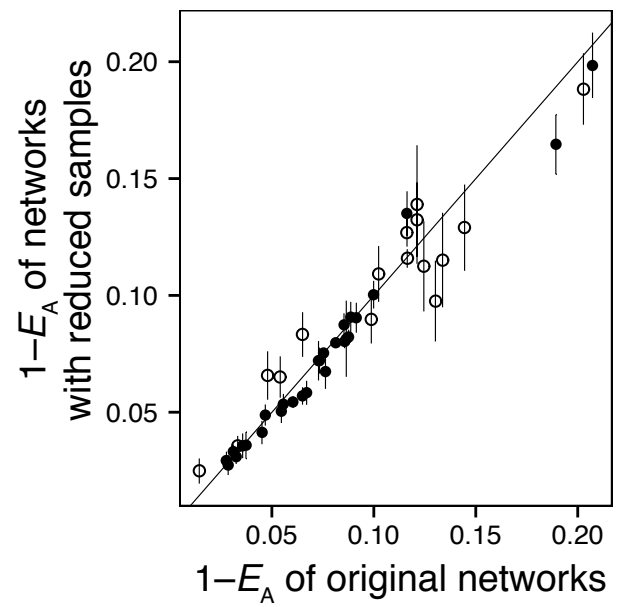

Supplement: Figure S2. (a) 1–EP and (b) 1–EA of the networks with reduced samples plotted against the corresponding values of the original networks [file rsos150630supp2.pdf]

Figure S3

(a)

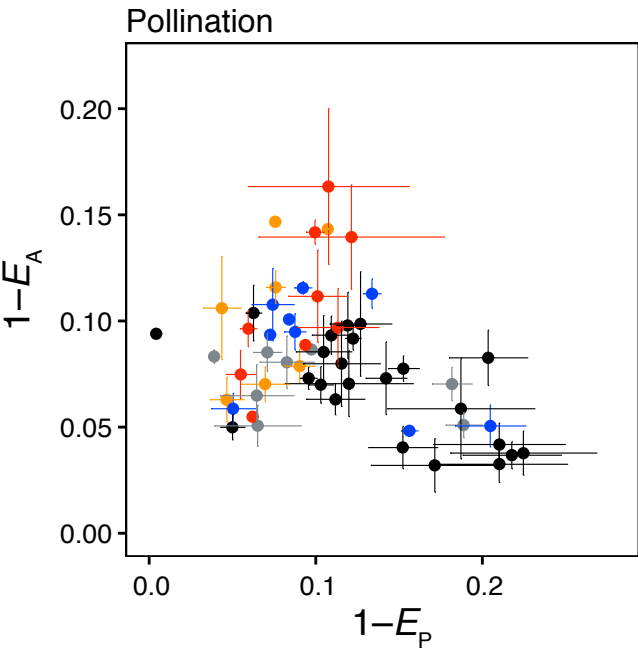

(b)

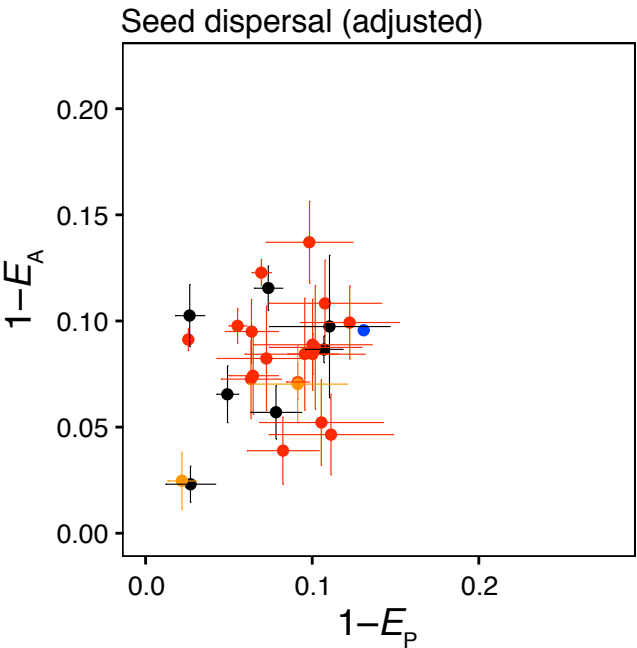

Supplement: Figure S3. Relationships between 1–EP and 1–EA for the networks with adjusted plant-animal ratio of (a) pollination and (b) seed dispersal. [file rsos150630supp3.pdf]

Figure S4

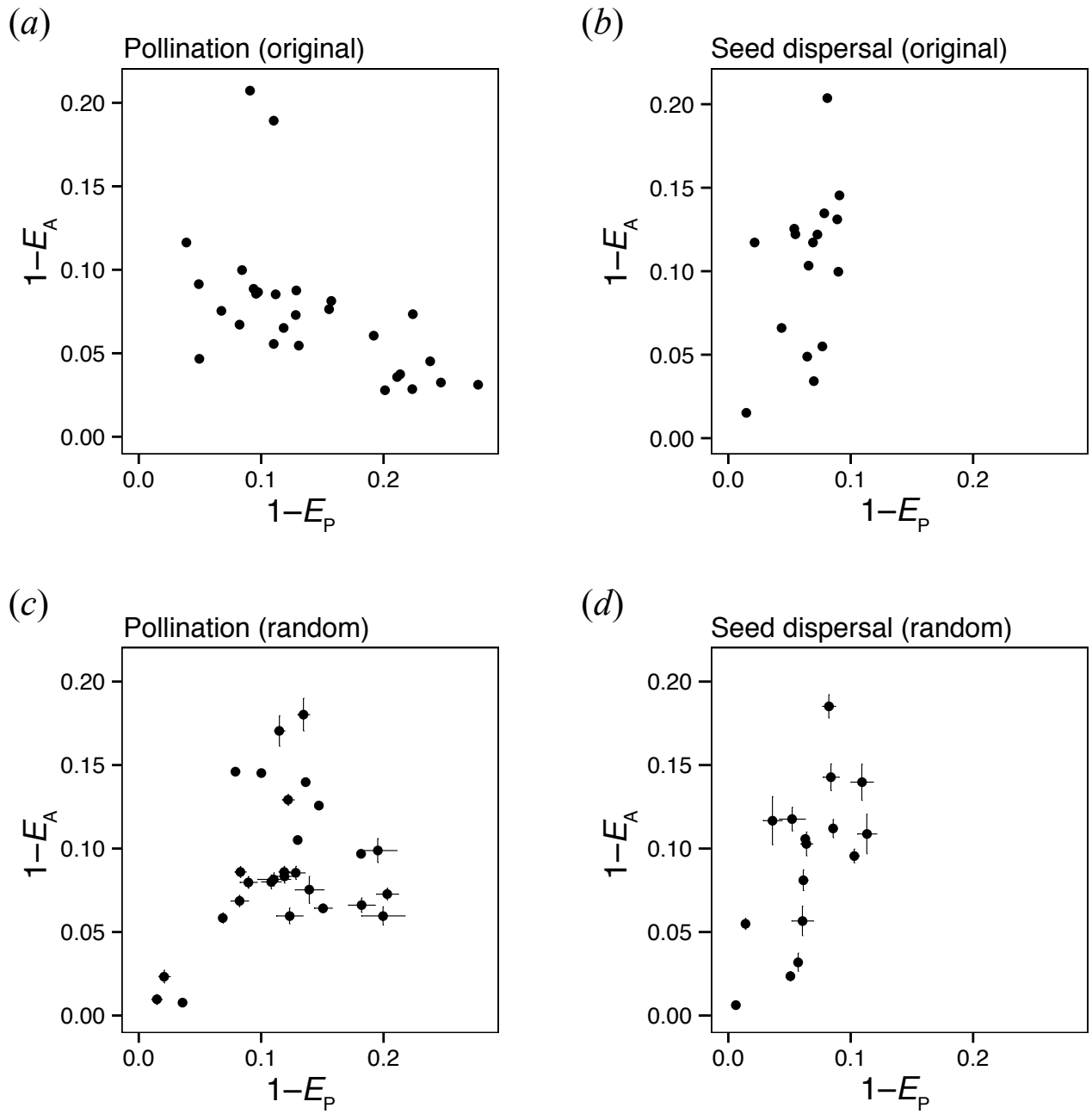

Supplement: Figure S4. Plots of 1–EP and 1–EA of original networks for (a) pollination and (b) seed dispersal and random networks for pollination (c) and (d) seed dispersal. [file rsos150630supp4.pdf]
