# Supplementary material for: Geographical variation in the heterogeneity of mutualistic networks
Source: R Soc Open Sci. 2016 Jun 8;3(6):150630. doi: 10.1098/rsos.150630 (PMC4929896; doi:10.1098/rsos.150630)
Supplement: Note S1. Comparison of observed and potential maximum values of 1–E. [file rsos150630supp5.pdf]

Comparison of observed and potential maximum values of 1–*E*

To examine the possibility that structural constraints such as low mean degrees per species limited potential ranges of 1–*E* and caused the patterns we observed, we compared observed 1–*E* with the potential maximum values. We calculated the potential maximum values of $1-E_P$ and $1-E_A$ independently for each network under these constraints that (1) the numbers of plant and animal species and the total number of links were equal to the original network, and that (2) all species had at least one link. Under the constraints, 1–*E* is maximized when some species have the maximum possible degree and the others have a degree of one. We also calculated the potential maximum values of the networks with animal-plant ratio adjusted (see the section Adjustment of plant-animal ratio in Materials and Methods).

We found that the observed values of 1–*E* were much smaller than the potential maximum values (figure SN1a, b). The potential maximum values of 1–*E* of plants and animals were negatively correlated for pollination, and positively correlated for seed-dispersal networks (figure SN1b) as found for the observed values (figure SN1a). For pollination, after adjustment of the plant-animal ratio (see the main text), the correlation of potential maximum values of 1–*E* between plants and animals was positive (figure SN1d), while that of the observed values was negative (figure SN1c). This suggests that the negative correlation between the potential maximum values is at least partly caused by the plant-animal ratio, while the negative correlation between the observed values is not. In seed dispersal, on the other hand, the relationships were always positive (figure SN1). The positive correlations in seed dispersal may therefore be caused by structural constraints of the networks.

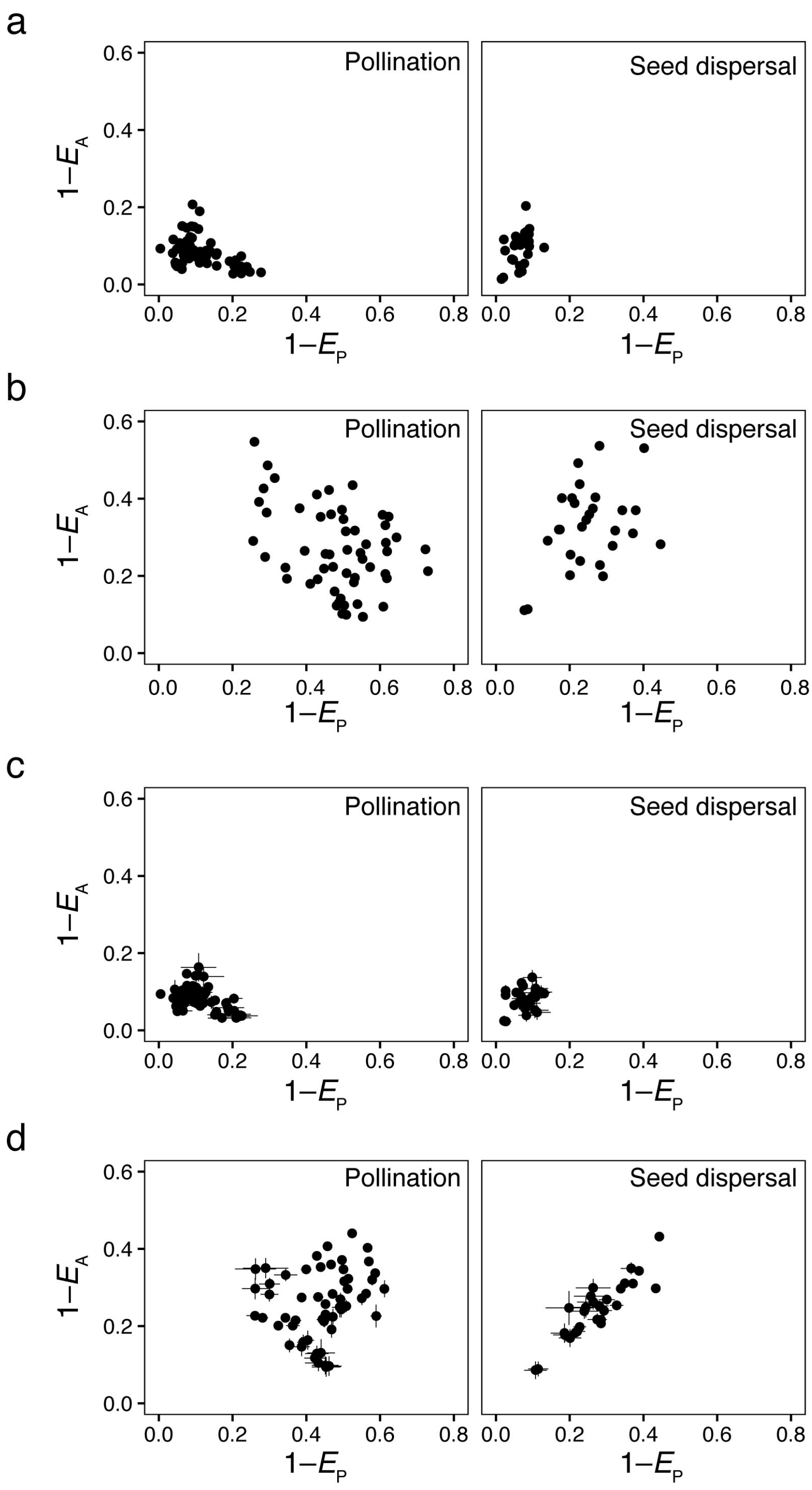

Figure SN1. Correlations between 1–E_P and 1–E_A. (a) The observed values (for pollination, $\rho = -0.45$, $p = 0.0005$, $n = 56$; for seed dispersal, $\rho = 0.43$, $p = 0.0221$, $n = 28$, Spearman Rank Correlation test). (b) The potential maximum values (for pollination, $\rho = -0.29$, $p = 0.0277$; for seed dispersal, $\rho = 0.21$, $p = 0.2779$, $n = 28$). (c) The observed values with the adjustment of the plant-animal ratio (for pollination, $\rho = -0.31$, $p = 0.0182$; for seed dispersal, $\rho = 0.19$, $p = 0.3401$, $n = 28$). (d) The potential maximum values with the adjustment of the plant-animal ratio (for pollination, $\rho = 0.28$, $p = 0.0338$; for seed dispersal, $\rho = 0.85$, $p < 0.0001$, $n = 28$).
