# Supplementary material for: Geographical variation in the heterogeneity of mutualistic networks
Source: R Soc Open Sci. 2016 Jun 8;3(6):150630. doi: 10.1098/rsos.150630 (PMC4929896; doi:10.1098/rsos.150630)
Supplement: Table S1. List of the 56 plant-pollinator networks and 22 plant-seed disperser network analyzed by this study. [file rsos150630supp6.pdf]

Table S1. List of the 56 plant-pollinator networks and 28 plant-seed disperser network analyzed by this study.

Pollination networks

Dataset[a]	Source[b]	Location	P[c]	A[d]	References	Region[e]
P1	B	Arctic tundra, Greenland	31	75	Elberling, H., and Olesen, J.M. Unpublished data.	A
**P2**	I	Latnjajaure, Sweden	23	118	Elberling, H., and Olesen, J.M. (1999). The structure of a high latitude plantflower visitor system: the dominance of flies. Ecography 22, 314-323.	A
P3	B	Ellesmere Island, N.W.T., Canada	29	81	Hocking, B. (1968). Insect flower associations in the high Arctic with special reference to nectar. Oikos 19, 359-387.	A
**P4**	O	Kibue, Kyoto, Japan	118	873	Inoue, T., Kato, M., Kakutani, T., Suka, T., and Itino, T. (1990). Insect flower relationship in the temperate deciduous forest of Kibune, Kyoto: An overview of the flowering phenology and the seasonal pattern of insect visits. Contrib. Biol. Lab. Kyoto Univ. 27, 377-463.	T
**P5**	I	Kosciusko National Park in the Snowy, Australia	36	81	Inouye, D.W., and Pyke, G.H. (1988). Pollination biology in the Snowy Mountains of Australia: comparisons with montane Colorado. Aust. J. Ecol. 13, 191-210.	M
**P6**	O	Kyoto University Campus, Japan	113	313	Kakutani, T., Inoue, T., Kato, M., and Ichihashi, H. (1990). Insect flower relationship in the campus of Kyoto University, Kyoto: An overview of the flowering phenology and the seasonal pattern of insect visits. Contrib. Biol. Lab. Kyoto Univ. 27, 465-521.	T
**P7**	B	Nakaikemi marsh, Tsuruga, Japan	64	187	Kato M., and Miura R. (1996). Flowering phenology and anthophilous insect community at a threatened natural lowland marsh at Nakaikemi in Tsuruga, Japan. Contrib. Biol. Lab. Kyoto Univ. 29, 1-48.	T
**P8**	O	Kyoto University Forest of Ashu, Japan	91	702	Kato, M., Kakutani, T., Inoue, T., and Itino, T. (1990). Insect flower relationship in the primary beech forest of Ashu, Kyoto: An overview of the flowering phenology and the seasonal pattern of insect visits. Contrib. Biol. Lab. Kyoto Univ. 27, 309-375.	T
**P9**	B	Mt. Kushigata, Shinsyu, Japan	90	354	Kato, M., Matsumoto, M., and Kato, T. (1993). Flowering phenology and anthophilous insect community in the cool-temperate subalpine forests and meadows at Mt. Kushigata in the Central part of Japan. Contrib. Biol. Lab. Kyoto Univ. 28, 119-172.	M
**P10**	I	Melville Island, N.W.T., Canada	11	18	Mosquin, T., and Martin, J. E. (1967). Observations on the pollination biology of plants on Melville Island, NWT Canada. Canad. Field Nat. 81, 201-205.	A
P11	B	Bog, Hestehaven, Denmark	10	40	Olesen. J.M. Unpublished data.	A
P12	B	Forest, Hestehaven, Denmark	8	42	Olesen. J.M. Unpublished data.	A
P13	B	Wasteground, Hestehaven, Denmark	28	82	Olesen. J.M. Unpublished data.	A
P15	B	Arthur's Pass, New Zealand	18	60	Primack, R.B. (1983). Insect pollination in the New Zealand mountain flora. New Zealand J. Bot. 21, 317-333.	I
P16	B	Cass, New Zealand	41	139	Primack, R.B. (1983). Insect pollination in the New Zealand mountain flora. New Zealand J. Bot. 21, 317-333.	I
P17	B	Craigieburn, New Zealand	49	118	Primack, R.B. (1983). Insect pollination in the New Zealand mountain flora. New Zealand J. Bot. 21, 317-333.	I
P19	B	Yufu, Kyusyu Island, Japan	99	293	Yamazaki, K., and Kato, M. (2003). Flowering phenology and anthophilous insect community inagrassland ecosystem at Mt. Yufu, Western Japan. Contrib. Biol. Lab. Kyoto Univ. 29, 255-318.	T
P20	I	Andean (alpine) zone, Cordon del Crepo, Chile	87	98	Arroyo, M.T.K., Primack, R.B., and Armesto, J.J. (1982). Community studies in pollination ecology in the high temperate Andes of Central Chile. I. Pollination mechanisms and altitudinal variation. Amer. J. Bot. 69, 82-97.	M
P21	I	Andean (alpine) zone, Cordon del Crepo, Chile	43	62	Arroyo, M.T.K., Primack, R.B., and Armesto, J.J. (1982). Community studies in pollination ecology in the high temperate Andes of Central Chile. I. Pollination mechanisms and altitudinal variation. Amer. J. Bot. 69, 82-97.	M

P22	I	Andean (alpine) zone, Cordon del Crepo, Chile	41	28	Arroyo, M.T.K., Primack, R.B., and Armesto, J.J. (1982). Community studies in pollination ecology in the high temperate Andes of Central Chile. I. Pollination mechanisms and altitudinal variation. Amer. J. Bot. 69, 82-97.	M
**P23**	I	New Brunswick, Canada	12	102	Barrett, S.C.H., and Helenurm, K. (1987). The Reproductive biology of boreal forest herbs I. Breeding systems and pollination. Can. J. Bot. 65, 2036-2046.	T
P24	I	Pikes Peak in Colorado, USA	96	276	Clements, R.E., and Long, F.L. (1923). Experimental pollination. An outline of the ecology of flowers and insects. Washington, D.C., USA, Carnegie Institute of Washington.	M
P25	I	Tenerife, Canary Islands	11	38	Dupont, Y.L., Hansen, D.M., and Olesen, J.M. (2003). Structure of a plant-flower-visitor network in the high-altitude sub-alpine desert of Tenerife, Canary Islands. Ecography 26, 301-310.	M
P26	I	Hazen Camp, Northern Ellesmere Island, Canadian Arctic Archipelago	32	115	Kevan, P.G. (1970). High arctic insect-flower visitor relations: the inter-relationships of arthropods and flowers at Lake Hazen, Ellesmere Island, Northwest Territories, Canada. Ph.D. thesis thesis, University of Alberta.	A
P28	I	Laguna, Diamante, Andes of Mendoza, Argentina	21	45	Medan, D., Montaldo, N.H., Devoto, M., Mantese, A., Vasellati, V., and Bartoloni, N.H. (2002). Plant-pollinator relationships at two altitudes in the Andes of Mendoza, Argentina. Arctic Antarctic and Alpine Res. 34, 233-241.	M
P29	I	Rio Branco, Andes of Mendoza, Argentina	23	72	Medan, D., Montaldo, N.H., Devoto, M., Mantese, A., Vasellati, V., and Bartoloni, N.H. (2002). Plant-pollinator relationships at two altitudes in the Andes of Mendoza, Argentina. Arctic Antarctic and Alpine Res. 34, 233-241.	M
**P30**	I	Bristol, UK	25	79	Memmott, J. (1999). The structure of a plant-pollinator food web. Ecol. Lett. 2, 276-280.	T
**P31**	I	Piedmont North Carolina, USA	13	44	Motten, A. F. (1986). Pollination ecology of the spring wildflower community of a temperate deciduous forest. Ecol. Monographs 56, 21-42.	T
**P32**	I	KwaZulu-Natal region, South Africa	9	56	Ollerton, J., Johnson, S.D., Cranmer, L., and Kellie, S. (2003). The pollination ecology of an assemblage of grassland asclepiads in South Africa. Ann. Bot. 92, 807-834.	T
P33	I	Central plains of Guarico State, Venezuela.	33	53	Ramirez, N. and Y. Brito. (1992). Pollination biology in a palm swamp community in the Venezuelan central plains. Bot. J. Linn. Soc. 110, 277-302.	R
**P34**	I	Mer Bleue peat bog of Ottawa, Canada	13	34	Small, E. (1976). Insect pollinators of the Mer Bleue peat bog of Ottawa. Can. Field Naturalist 90, 22-28.	T
**P35**	I	AG, Nahuel Huapi National Park and surrounding areas in Rio Negro, Argentina	10	29	Vazquez, D.P. (2002). Interactions among introduced ungulates, plants, and pollinators: A field Study in the temperate forest of the Southern Andes. Doctoral Dissertation thesis, University of Tennessee, Knoxville, Tennessee, USA.	T
**P36**	I	CL, Nahuel Huapi National Park and surrounding areas in Rio Negro, Argentina	9	33	Vazquez, D.P. (2002). Interactions among introduced ungulates, plants, and pollinators: A field Study in the temperate forest of the Southern Andes. Doctoral Dissertation thesis, University of Tennessee, Knoxville, Tennessee, USA.	T
**P37**	I	LL, Nahuel Huapi National Park and surrounding areas in Rio Negro, Argentina	9	27	Vazquez, D.P. (2002). Interactions among introduced ungulates, plants, and pollinators: A field Study in the temperate forest of the Southern Andes. Doctoral Dissertation thesis, University of Tennessee, Knoxville, Tennessee, USA.	T
**P38**	I	MH, Nahuel Huapi National Park and surrounding areas in Rio Negro, Argentina	10	29	Vazquez, D.P. (2002). Interactions among introduced ungulates, plants, and pollinators: A field Study in the temperate forest of the Southern Andes. Doctoral Dissertation thesis, University of Tennessee, Knoxville, Tennessee, USA.	T
**P39**	I	MNH, Nahuel Huapi National Park and surrounding areas in Rio Negro, Argentina	8	35	Vazquez, D.P. (2002). Interactions among introduced ungulates, plants, and pollinators: A field Study in the temperate forest of the Southern Andes. Doctoral Dissertation thesis, University of Tennessee, Knoxville, Tennessee, USA.	T

**P40**	I	QH, Nahuel Huapi National Park and surrounding areas in Rio Negro, Argentina	8	26	Vazquez, D.P. (2002). Interactions among introduced ungulates, plants, and pollinators: A field Study in the temperate forest of the Southern Andes. Doctoral Dissertation thesis, University of Tennessee, Knoxville, Tennessee, USA.	T
**P42**	I	SL, Nahuel Huapi National Park and surrounding areas in Rio Negro, Argentina	8	27	Vazquez, D.P. (2002). Interactions among introduced ungulates, plants, and pollinators: A field Study in the temperate forest of the Southern Andes. Doctoral Dissertation thesis, University of Tennessee, Knoxville, Tennessee, USA.	T
**P43**	I	Flores, Azorean forest, Macronesia	14	13	Olesen, J.M., Eskildsen, L.I., and Venkatasami, S. (2002). Invasion of pollination networks on oceanic islnds: importance of invader complexes and endemic super generalists. Diversity and Distributions, 8, 181-192.	I
**P44**	I	Ile aux Aigrettes, Mauritius	10	12	Olesen, J.M., Eskildsen, L.I., and Venkatasami, S. (2002). Invasion of pollination networks on oceanic islnds: importance of invader complexes and endemic super generalists. Diversity and Distributions, 8, 181-192.	I
**P45**	O	Lambir Hills, Borneo	41	33	Kato, M. (1996). Plant pollinator interactions in the understory of a lowand mixed dipterocarp forest in Sarawak. Amer. J. Bot. 83: 732-743.	R
**P46**	O	Reserva de Cerrado de Corumbataí, Brazil	66	103	Andena, S.R., Bego, L.R., and Mechi, M.R. (2005). A Comunidade de abelhas (Hymenoptera, Apoidea) de uma área de cerrado (Corumbataí, SP) e suas visitas às flores. Res. Bras. Zoociências Juiz de Flora 7, 55-91. (Plant species in Cypelaceae were removed from the analysis)	R
P47	O	Chã-Grande, Agreste de Pernambuco, Brasil	72	78	Milet-Pinheiro, P., and Schlindwein, C. (2008). Comunidade de abelhas (Hymenoptera, Apoidea) e plantas em uma área do Agreste pernambucano, Brasil. Revista Brasileira de Entomologia 52, 625-636.	R
P48	I	Carlinville, Illinois, USA	456	1044	Robertson, C. (1929). Flowers and insects: lists of visitors to four hundred and fifty-three flowers. Carlinville, IL, USA.	T
P49	R	Canaima Nat. Park, Venezuela	48	49	Ramirez, N. (1989). Biología de polinización en una comunidad arbustiva tropical de la alta Guyana Venezolana. Biotropica 21, 319-330.	R
P50	R	Chiloe, Chile	26	128	Smith-Ramírez C., Martinez, P., Nuñez, M., González, C., and Armesto J.J. (2005). Diversity, flower visitation frequency and generalism of pollinators in temperate rain forests of Chiloé Island, Chile. Bot. J. Linn. Soc. 147, 399-416.	T
**P51**	R	Hickling, Norfolk, UK	17	61	Dicks, L.V., Corbet, S.A., and Pywell, R.F. (2002). Compartmentalization in plant–insect flower visitor webs. J. Anim. Ecol. 71, 32-43.	T
**P52**	R	Shelfanger, Norfolk, UK	16	36	Dicks, L.V., Corbet, S.A., and Pywell, R.F. (2002). Compartmentalization in plant–insect flower visitor webs. J. Anim. Ecol. 71, 32-43.	T
P53	R	Morant Point, Jamaica	61	36	Percival, M. (1974). Floral ecology of coastal scrub in sotheast Jamaica. Biotropica 6, 104-129.	R
P54	I	Itatim, Bahia State, Northeastern Brazil	51	25	Santos, G.M.M, Aguiar, C.M.L., and Mello, M.A.R. (2010). Flower-visiting guild associated with the Caatinga flora: trophic interaction networks formed by social bees and social wasps with plants. Apidologie 41, 466-475.	R
P55	R	Daphní, Athens, Greece	131	666	Petanidou, T. (1991). Pollination ecology in a phryganic ecosystem. PhD. Thesis, Aristotelian University, Thessaloniki.	T
P56	I	Restored site, Petrin, Mauritius Island	73	135	Kaiser-Bunbury, C.N., Muff, S., Memmot, J., Muller, C.B., and Caflisch, A. (2010). The robustness of pollination networks to the loss of species and interactions: a quantitative approach incorporating pollinator behaviour. Ecol. Lett. 13, 442-452.	I
P57	I	Control site, Petrin, Mauritius Island	58	100	Kaiser-Bunbury, C.N., Muff, S., Memmot, J., Muller, C.B., and Caflisch, A. (2010). The robustness of pollination networks to the loss of species and interactions: a quantitative approach incorporating pollinator behaviour. Ecol. Lett. 13, 442-452.	I

P58	R	Reserva Biologica de Donana	26	179	Herrera, J. (1988). Pollination relatioships in southern spanish mediterranean shrublands. J. Ecol. 76, 274-287.	T
**P59**	O	Western Kenya	65	37	Hagen, M., and Kraemer, M. (2010). Agricultural surroundings support flower–visitor networks in an Afrotropical rain forest. Biol. Conserv. 143, 1654-1663.	R
**P60**	O	Western Kenya	90	35	Hagen, M., and Kraemer, M. (2010). Agricultural surroundings support flower–visitor networks in an Afrotropical rain forest. Biol. Conserv. 143, 1654-1663.	R

Seed dispersalr networks

Dataset[a]	Source[b]	Location	P[c]	A[d]	References	Region[f]
**SD1**	I	Mount Missim, north-northeast of Wau, Papua New Guinea	31	9	Beehler, B. (1983). Frugivory and polygamy in birds of paradise. Auk 100, 1-12.	T
**SD2**	I	Wytham woods, near Oxford, South Britain	11	14	Sorensen, A.E. (1981). Interactions between birds and fruit in a temperate woodland. Oecologia 50, 242-249.	T
SD3	B	Mtunzini, South Africa	17	9	Frost, P.G.H. (1980). Fruit-frugivore interactions in a South African coastal dune forest. In: Acta XVII Congressus Internationalis Ornithologici (ed. Noring, R.), pp. 1179-1184. Deutsche Ornithologische Ges., Berlin, Germany.	T
SD6	B	Mediterranean montane forest, Cazorla, Spain.	25	33	Jordano, P. Unpublished data.	M
SD7	B	Hato Raton, Donana, Spain.	16	17	Jordano, P. (1985). El ciclo anual de los paseriformes frugívoros en el matorral mediterráneo del sur de España: importancia de su invernada y variaciones interanuales. Ardeola, 32, 69-94.	T
**SD8**	I	Barro Colorado Island and Soberania National Park, Panama	13	11	Poulin, B.,Wright, S.J., Lefebvre, G., and Calderon, O. (1999). Interspecific synchrony and asynchrony in the fruiting phenologies of congeneric bird-dispersed plants in Panama. J.Trop. Ecol. 15, 213-227.	R
**SD9**	I	Arima Valley, Trinidad	65	14	Snow, B.K., and Snow, D.W. (1971). The feeding ecology of tanagers and honeycreepers in Trinidad. Auk 88, 291-322.	R
**SD10**	I	Calton, England	29	19	Snow, B.K., and Snow, D.W. (1988). Birds and Berries. Calton, England.	T
**SD12**	R	Caguana, Puerto Rico	25	16	Carlo, T.A., Collazo, J.A., and Groom, M.J. (2003). Avian fruit preferences across a Puerto Rican forested landscape: pattern consistency and implications for seed removal. Oecologia 134, 119-131.	R
**SD13**	R	Cialitos, Puerto Rico	34	20	Carlo, T.A., Collazo, J.A., and Groom, M.J. (2003). Avian fruit preferences across a Puerto Rican forested landscape: pattern consistency and implications for seed removal. Oecologia 134, 119-131.	R
**SD14**	R	Cordillera, Puerto Rico	25	13	Carlo, T.A., Collazo, J.A., and Groom, M.J. (2003). Avian fruit preferences across a Puerto Rican forested landscape: pattern consistency and implications for seed removal. Oecologia 134, 119-131.	R
**SD15**	R	Fronton, Puerto Rico	21	15	Carlo, T.A., Collazo, J.A., and Groom, M.J. (2003). Avian fruit preferences across a Puerto Rican forested landscape: pattern consistency and implications for seed removal. Oecologia 134, 119-131.	R
SD17	R	North Negros Forest Reserve, Central Philippine Islands	36	19	Hammann, A. and Curio, B. (1999). Interactions among frugivores and fleshy fruit trees in a Philippine submontane rainforest. Conserv. Biol. 13, 766-773.	R
SD20	R	Kuala Lompat, Krau Game Reserve	25	61	Lambert, F. (1989). Fig-eating by birds in a Malaysian lowland rain forest. J. Trop. Ecol. 5, 401-412.	R
SD21	R	Crater Mountain Biological Research Station, Papua New Guinea	29	32	Mack, A.L., and Wright, D.D. (1996). Notes on occurrence and feeding of birds at Crater Mountain Biological Research Station, Papua New Guinea. Emu 96, 89-101.	R
SD22	R	Monteverde, Costa Rica	169	40	Wheelwright, N.T., Haber, W.A., Murray, K.G., and Guindon, C. (1984). Tropical fruit-eating birds and their food plants: a survey of a Costa Rican lower montane forest. Biotropica 16, 173-192.	R
SD23	R	Yakushima Island, Japan	15	8	Noma, N., and Yumoto, T. (1997). Annual fluctuations of sapfruits production and synchronization within and inter species in a warm temperate forest on Yakushima Island, Japan. Tropics 6, 441-449.	T

SD24	R	Intervales and Saibadela, São Paulo, Brazil	207	110	Silva, W.R., Marco, P. De, Hasui, E., and Gomes, V.S.M. (2002). Patterns of fruit-frugivores interactions in two Atlantic Forest bird communities of South-eastern Brazil: implications for conservation. Pp. 423-435. In: Levey, D.J., Silva, W.R., and Galetti, M. (eds.) Seed dispersal and frugivory: ecology, evolution and conservation. Wallinford, CAB International.	T
**SD25**	R	Santa Genebra Reserve, SE Brazil	35	29	Galetti, M., and Pizo, M.A. (1996). Fruit eating birds in a forest fragment in southeastern Brazil. Ararajuba, Revista Brasileira de Ornitologia 4, 71-79.	T
**SD26**	I	Kakamega Forest, Kenya	33	88	Schleuning, M., Blüthgen, N., Flörchinger, M., Braun, J., Schaefer, H.M., and Böhning-Gaese, K. (2011). Specialization and interaction strength in a tropical plant-frugivore network differ among forest strata. Ecology 92, 26-36.	R
**SD30**	O	La Selva, Costa Rica	35	14	Lopez, J.E. and Vaughan, C. (2007). Food niche overlap among neotropical frugivorous bats in Costa Rica. Revista de Biología Tropical 55, 301 -313.	R
**SD31**	O	Centro de Investigaciones Jerano Herrera, Province of Requena, Peru	61	18	Gorchov, D.L., Cornejo, F., Ascorra, C.F., and Jaramillo, M. (1995). Dietary overlap between frugivorous birds and bats in the Peruvian Amazon. Oikos 74, 235-250.	R
SD32	O	Hesse, Germany	30	31	Stiebel, H. and Bairlein, F. (2008). Frugivory in central European birds I: Diet selection and foraging. Vogelwarte 46, 1-23.	T
**SD33**	O	Galapagos Islands	58	18	Heleno, R.H., Olesen, J.M., Nogales, M., Vargas, P. and Traveset, A. (2013). Seed dispersal networks in the Galá pagos and the consequences of alien plant invasions. Proc. Roy. Soc. B. 280, 2012-2112.	I
SD34	O	Nhecolândia, Brazil	45	46	Donatti, C.I., Guimarães, P.R., Galetti, M., Pizo, M.A., Marquitti, F.M.D., Dirzo, R. (2011). Analysis of a hyper-diverse seed dispersal network: modularity and underlying mechanisms. Ecol. Lett.14, 773-781.	R
**SD35**	M	Reserva de Santa Genebra, Brazil	10	34	Faria D.M., (1996) Uso de recursos alimentares por morcegos filostomídeos fitófagos na Reserva de Santa Genebra, Campinas, São Paulo. MSc Dissertation. Universidade Estadual de Campinas.	R
SD36	M	Barro Colorado Island, Panama	11	47	Giannini, N.P., Kalko, E.K.V. (2004). Trophic structure in a large assemblage of phyllostomid bats in Panama. Oikos 105, 209-220.	R
**SD37**	O	Zealandia, North Island of New Zealand	11	37	Burns, K. C. (2013) What causes size coupling in fruit–frugivore interaction webs? Ecology 94, 295-300.	I

[a]Bold characters indicate datasets with quantitative data.

[b]Source: B, Bascompte et al. (2003) [1]; I, Interaction Web Database [2]; M, Mello et al.[3]; R, Rezende et al. (2007), [4]; O, from original literature.

[c]Number of plant species included.

[d]Number of animal species included.

[e] A: arctic and boreal (latitude > 55°), T: temperate (23° < latitude < 55°, altitude <1600 m), M: montane (23° < latitude < 55°, altitude >1600 m), R: tropics and subtropics (latitude < 23°), I:

Reference for Table S1

1. Bascompte, J., Jordano, P. & Olesen, J. M. 2006 Asymmetric coevolutionary networks facilitate biodiversity maintenance. Science 312, 431–3. (doi:10.1126/science.1123412)
2. Interaction Web Database (http://www.nceas.ucsb.edu/interactionweb/index.html)
3. Mello, M. A. R., Marquitti, F. M. D., Guimarães, P. R., Kalko, E. K. V., Jordano, P. & de Aguiar, M. A. M. 2011 The missing part of seed dispersal networks: Structure and robustness of bat-fruit interactions. PLoS One 6, 1–10. (doi:10.1371/journal.pone.0017395)
4. Rezende, E. L., Lavabre, J. E., Guimarães, P. R., Jordano, P. & Bascompte, J. 2007 Non-random coextinctions in phylogenetically structured mutualistic networks. Nature 448, 925–8. (doi:10.1038/nature05956)
