# Supplementary material for: Geographical variation in the heterogeneity of mutualistic networks
Source: R Soc Open Sci. 2016 Jun 8;3(6):150630. doi: 10.1098/rsos.150630 (PMC4929896; doi:10.1098/rsos.150630)
Supplement: Table S2. Four evenness indices that are robust to changes in rare species and that respond moderately to changes in abundance of median and dominant species identified by Beisel [21]. [file rsos150630supp7.pdf]

Table S2. Four evenness indices that are robust to changes in rare species and that respond moderately to changes in abundance of median and dominant species identified by Beisel [1].

Code	Formulae	Reference
E_{Pielou} [a]	$\frac{-\sum_{i=1}^{S} p_i \cdot \ln(p_i)}{\ln(S)}$	[3]
E_{1-D} [a]	$\frac{1-\sum_{i=1}^{S} (p_i)^2}{1-1/S}$	[2]
$E_{-\ln D}$	$\frac{-\ln \sum_{i=1}^{S} (p_i)^2}{-\ln(S)}$	[2]
E_{MI}	$\frac{1-\sqrt{\sum_{i=1}^{S} (p_i)^2}}{1-1/\sqrt{S}}$	[4]

[a]To calculate E_{1-D} and $E_{-\ln D}$, the original definition of Simpson's dominance index, $D=\sum_{i=1}^{S} (p_i)^2$, rather than the modified version for a finite community, $D=\frac{\sum_{i=1}^{S} (n_i)^2 \cdot (n_i-1)}{N \cdot (N-1)}$ was used to restrict the upper limits of the variables to 1, following Smith and Wilson [2]. *S*, number of plant/animal species; p_i, proportion of links belonging to species *i, N*, total number of links; n_i, total number of links belonging to species *i*.

References for Table S2

1. Beisel, J. 2003 A comparative analysis of evenness index sensitivity. *Int. Rev. ...* **88**, 3–15. (doi:10.1002/iroh.200390004)

2. Smith, B. & Wilson, J. B. 1996 A consumer's guide to evenness indices. *Oikos* **76**, 70–82. (doi:10.2307/3545749)

3. Pielou, E. C. 1967 The measurement of diversity in different types of biological collections. *J. Theor. Biol.* **15**, 177. (doi:10.1016/0022-5193(67)90048-3)

4. Mcintosh, R. P. 1967 And index of diversity and the relation of certain concepts to diversity. *Ecology* **48**, 392–404. (doi:10.2307/1932674)
